# Host-dependent C-to-U RNA editing in SARS-CoV-2 creates novel viral genes with optimized expressibility

**DOI:** 10.3389/fcimb.2024.1476605

**Published:** 2024-10-09

**Authors:** Pirun Zhang, Wenli Zhang, Jiahuan Li, Huiying Liu, Yantong Yu, Xiaoping Yang, Wenqing Jiang

**Affiliations:** ^1^ The Second Institute of Clinical Medicine, Guangzhou University of Chinese Medicine, Guangzhou, Guangdong, China; ^2^ Qingdao Mental Health Center, Qingdao, Shandong, China; ^3^ Qingdao Central Hospital, University of Health and Rehabilitation Sciences (Qingdao Central Hospital), Qingdao, Shandong, China; ^4^ Qingdao Hospital of Traditional Chinese Medicine, Qingdao Haici Hospital, Qingdao, Shandong, China; ^5^ Pulmonary and Critical Care Medicine Department 2, Qingdao Hiser Hospital Affiliated of Qingdao University (Qingdao Traditional Chinese Medicine Hospital), Qingdao, Shandong, China; ^6^ College of First Clinical Medical, Shandong University of Traditional Chinese Medicine, Jinan, Shandong, China

**Keywords:** SARS-CoV-2, C-to-U RNA editing, novel genes, TAI, positive selection

## Abstract

Rampant C-to-U RNA editing drives the mutation and evolution of SARS-CoV-2. While much attention has been paid to missense mutations, the C-to-U events leading to AUG and thus creating novel ORFs were uninvestigated. By utilizing the public time-course mutation data from the worldwide SARS-CoV-2 population, we systematically identified the “AUG-gain mutations” caused by C-to-U RNA editing. Synonymous mutations were of special focus. A total of 58 synonymous C-to-U sites are able to create out-of-frame AUG in coding sequence (CDS). These 58 synonymous sites showed significantly higher allele frequency (AF) and increasing rate (*d*AF/*d*t) than other C-to-U synonymous sites in the SARS-CoV-2 population, suggesting that these 58 AUG-gain events conferred additional benefits to the virus and are subjected to positive selection. The 58 predicted new ORFs created by AUG-gain events showed the following advantages compared to random expectation: they have longer lengths, higher codon adaptation index (CAI), higher Kozak scores, and higher tRNA adaptation index (tAI). The 58 putatively novel ORFs have high expressibility and are very likely to be functional, providing an explanation for the positive selection on the 58 AUG-gain mutations. Our study proposed a possible mechanism of the emergence of *de novo* genes in SARS-CoV-2. This idea should be helpful in studying the mutation and evolution of SARS-CoV-2.

## 1 Introduction

### 1.1 C-to-U RNA editing drives the continuous mutation and evolution of global SARS-CoV-2

A major concern on the COVID-19 pandemic is the endless mutations in the SARS-CoV-2 sequences (Liu et al., 2022). Although the basic evolutionary theory (Kimura, 1979) tells us that most novel mutations in SARS-CoV-2 might not increase the fitness of the virus, the public concern could not be eased. Once an adaptive mutation occurs in the SARS-CoV-2 population, it will be positively selected and then the strain(s) carrying this mutation would rapidly become the dominant strain globally.

Intriguingly, the rampant mutation of SARS-CoV-2 is introduced by the host ourselves (Simmonds, 2020; Zhang et al., 2021; Zhao et al., 2022; Li et al., 2023). C-to-U RNA editing is ubiquitous in plants and animals (Chu and Wei, 2019; Duan et al., 2023a; Duan et al., 2023d), modifying endogenous RNAs as well as invading viral RNAs. So far, it is commonly believed that the C-to-U RNA editing events (Simmonds, 2020; Liu et al., 2022) rather than RNA-replication errors (Di Giorgio et al., 2020; Zong et al., 2022) are the major source of SARS-CoV-2 mutations. C-to-U RNA editing in SARS-CoV-2 is inevitably exerted by APOBECs in host cells and the frequency of C-to-U editing is remarkably higher than that of other mutation types (Liu et al., 2022; Li et al., 2023).

Under the continuously extensive C-to-U RNA editing, a viral sequence would mutate and might accidentally acquire higher transmissibility or virulence (or both), the consequence of which will be the prevalence of this advantageous strain. At the molecular level, the fitness (including transmissibility and virulence) of the virus is connected to the genomic mutations that affect the *cis*-regulatory elements in SARS-CoV-2 sequence (Wang et al., 2021; Zhang et al., 2022; Zhu et al., 2022). *Cis* elements control the expression of viral genes, and the abundance of viral proteins directly determine the phenotypical behavior of the virus.

### 1.2 Gene expressibility: regulation and natural selection: on transcription and translation

According to the central dogma of molecular biology (Crick, 1970), the protein abundance of coding genes depends on the rates of transcription and translation. Both biological processes are associated with *cis*-regulatory elements. It was reported that the transcript abundance of genes was highly correlated with synonymous codon usage bias (SCUB) (Gouy and Gautier, 1982; Sharp et al., 1986) and that the translation rate was largely determined by Kozak sequence (translation initiation) (Ambrosini et al., 2022) and tRNA concentration (translation elongation) (Varenne et al., 1984; Dana and Tuller, 2014). Accordingly, three parameters, named codon adaptation index (CAI) (Sharp and Li, 1987), Kozak score (Gleason et al., 2022), and tRNA adaptation index (tAI) (dos Reis et al., 2004), were invented to measure the synonymous codon optimality, Kozak sequence, and tRNA availability of a gene, respectively.

We define gene expressibility as the collective effects and consequences of gene transcription and translation. Intuitively, mutations that enhance the CAI, Kozak score, or tAI would potentially elevate the expressibility of a gene. These optimal mutations are advantageous and should be favored by natural selection. In fact, the selection on gene expressibility has already been reported in SARS-CoV-2. For example, the selection on synonymous codon usage was reflected at both inter-species scale and intra-species scale of SARS-CoV-2 (Li et al., 2020a; Yu et al., 2021; Zhang et al., 2021). The mutations that increased the viral translation were also positively selected (Wang et al., 2021; Zhang et al., 2022; Zhu et al., 2022).

### 1.3 Another way to optimize gene expressibility: *de novo* gene emerging from existing genome

The abovementioned ways to increase the gene expressibility are based on the classic “evolution and tinkering” theory (Jacob, 1977) that describes the gradual amendment and optimization of genome sequences. However, optimized gene sequences could also be created by innovation (Jablonska and Tawfik, 2022). Basically, the prerequisite of a coding sequence (CDS) is a start codon (AUG) that can be properly recognized by the scanning ribosome. If a mutation creates an AUG within a suitable context, this AUG might capture the ribosome and initiate translation. However, most newly created AUGs are non-functional and are rapidly eliminated by purifying selection. Only very few new AUGs are able to initiate a functional gene (with potentially high gene expressibility). The functional AUGs will eventually become real start codons. This mechanism does not require a long-term tinkering on existing CDS. Instead, it appears to be an accidental gain of a novel gene with already optimized sequences. Then, the mutations that create such functional AUGs would be positively selected, exhibiting higher allele frequency (AF) in the population or even be fixed in a species.

**Figure 1:**
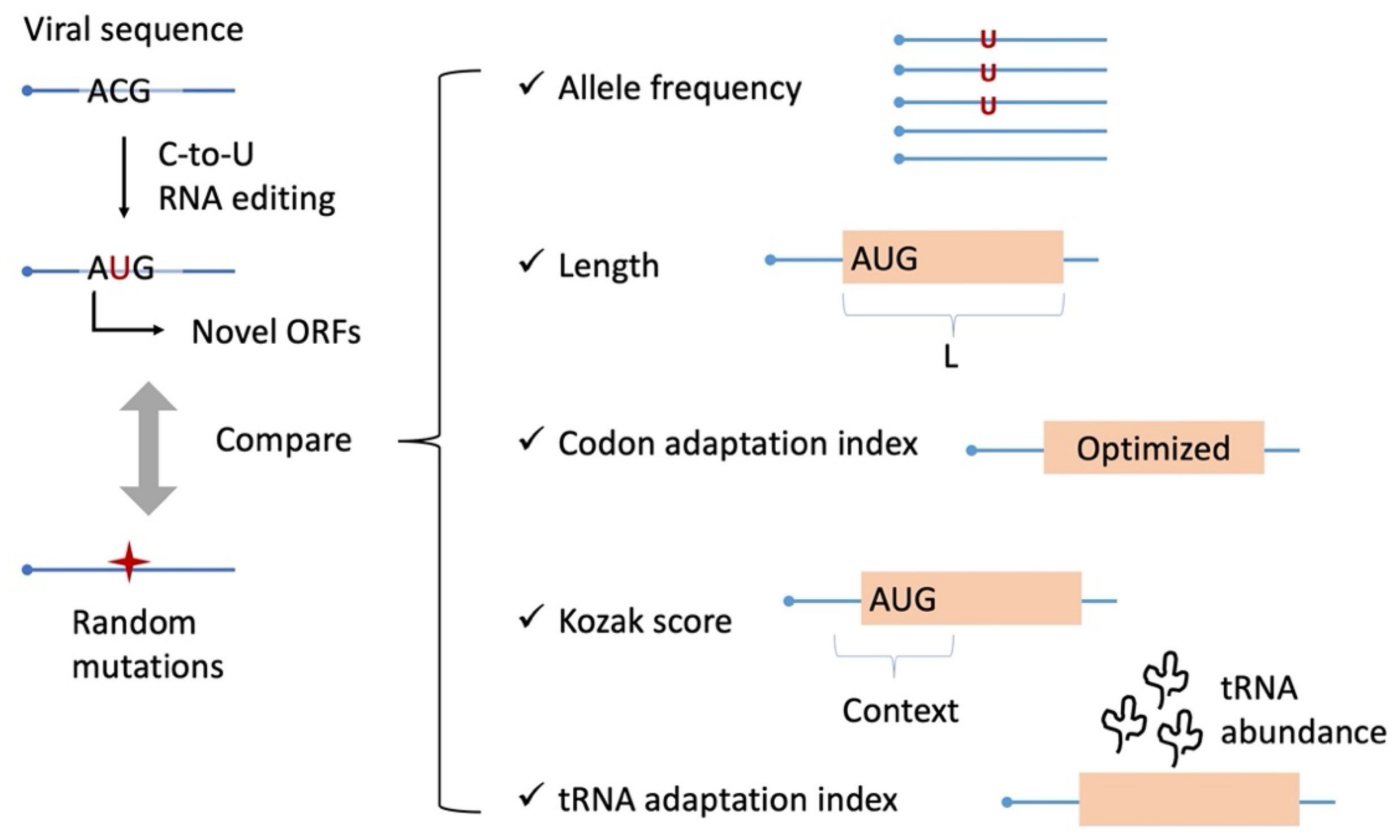
Scheme of this study. By systematic identification of AUG-gain mutations, we compare them with random mutations as a control. Different aspects of the mutation itself and the ORF created by this mutation are compared.

In this study, by utilizing the public time-course mutation data from the worldwide SARS-CoV-2 population (Zhu et al., 2022; Li et al., 2023), we systematically identified the “AUG-gain mutations” caused by C-to-U RNA editing (**Figure 1**). Notably, there were 58 synonymous C-to-U sites that created out-of-frame AUG in CDS. These 58 synonymous sites showed significantly higher AF and *d*AF/*d*t than other synonymous sites in the SARS-CoV-2 population, suggesting that these 58 AUG-gain events conferred additional benefits to the virus (compared to other normal synonymous sites). Strikingly, the 58 potential new ORFs created by AUG-gain events showed the following adaptive signals compared to random expectation: longer lengths, higher CAI, higher Kozak scores, and higher tAI. These results indicate that the 58 potential novel ORFs have high expressibility and are very likely to be functional, providing an explanation for positive selection on the 58 AUG-gain mutations that created these ORFs. Our study proposed a possible mechanism of the emergence of *de novo* genes in SARS-CoV-2. This idea should be helpful in studying the mutation and evolution of SARS-CoV-2.

## 2 Methods

### 2.1 Data collection

We downloaded the time-course mutation profile of worldwide SARS-CoV-2 sequences from the supplementary data of a previous study (Zhu et al., 2022). The data of that original study were produced using the public SARS-CoV-2 data from GISAID (Shu and McCauley, 2017). In the time-course study (Zhu et al., 2022), data from 16 time points were collected from 1 July 2021 to 15 February 2022, with equal time intervals of 15 days. The collection is performed before the emergence of Omicron and thus smartly avoids the bias caused by the rapid spread of Omicron.

### 2.2 Calculation of two parameters: correlation and slope

For each mutation site in SARS-CoV-2, the derived allele frequency (DAF) of 16 time points were available (Li et al., 2023). Slope is defined as d*AF*/d*t* (Li et al., 2023) and the Spearman’s correlation coefficient of the DAF against the 16 time points. Since the 16 time points are equally distributed, there is no essential difference between Spearman correlation and Pearson correlation.

### 2.3 Codon adaptation index

To define CAI, we first need to define relative synonymous codon usage (RSCU) (Sharp and Li, 1987). RSCU is the relative frequency of a codon among the total number of all its synonymous codons. For example, Lys has two codons, AAA and AAG; if, in the whole genome, there are 400 AAA codons and 600 AAG codons, then the RSCU for AAA is 0.8 and the RSCU for AAG is 1.2. The sum of RSCU of all synonymous codons is equal to the total number of synonymous codons of that amino acid. Then, the CAI of each ORF is the geometric mean of the RSCU values of all codons in that ORF.

### 2.4 tRNA adaptation index

tAI is similar to CAI; the difference is that tAI is not the geometric mean of RSCU, but the geometric mean of a parameter called wij. The wij for each codon is determined by the tRNA copy numbers in the genome (dos Reis et al., 2004), which could be understood as the weighted sum of tRNAs that decode this codon. For each codon, higher wij correlates to higher decoding rate and thus faster translation elongation rate. Higher tAI for a gene correlates to higher overall translation rate.

### 2.5 Kozak sequence and Kozak score

Kozak sequence refers to the 10-bp region from the –6 to +4 positions around the start codon AUG (where A is the +1 position) and Kozak score is widely used to measure the optimality of translation initiation of an ORF (Hata et al., 2021; Gleason et al., 2022). Note that the connection between Kozak sequence and translation initiation rate is mediated by the translation machinery of the cellular system, and optimal Kozak sequences are favored by translation machineries like ribosomes. Since SARS-CoV-2 genes depend on host (human) cells to translate, the optimality of the viral Kozak sequence should be judged based on the global human genes. To do so, a position-weighted matrix of the 10-bp Kozak sequence is generated from the human reference genome, and then each SARS-CoV-2 gene is assigned by a Kozak score according to its Kozak sequence. Higher Kozak scores represent potentially higher translation rates in human cells.

## 3 Results

### 3.1 C-to-U RNA editing is able to create internal AUG in the CDS of SARS-CoV-2

**Figure 2:**
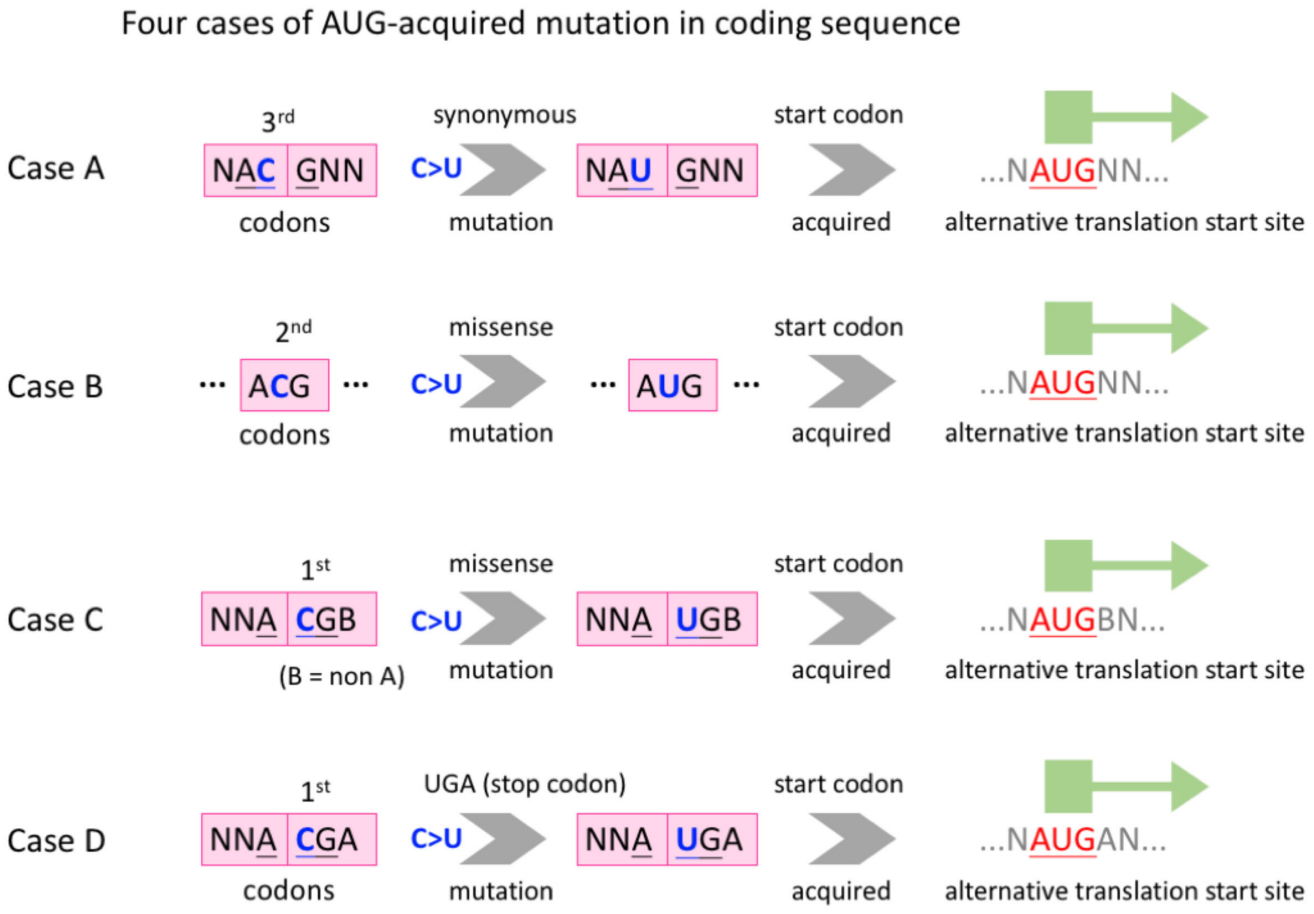
Definition of four cases where C-to-U RNA editing creates an internal AUG in CDS. Case **(A)**, a synonymous C>U mutation at the third codon position. Case **(B)**, a missense C>U mutation at the second codon position. Cases **(C, D)**, a missense C>U mutation at the first codon position. The difference between C and D is that whether the focal codon is a sense codon or a stop codon after C>U.

We collected the time-course mutation profile of worldwide SARS-CoV-2 sequences from a previous study (Zhu et al., 2022), the data of which were originally produced using the public SARS-CoV-2 data from GISAID (Shu and McCauley, 2017). The description of the collected data will be mentioned later, and here we propose the following four cases where C-to-U RNA editing creates an internal AUG in CDS (**Figure 2**).

Case A, a synonymous C>U mutation, occurs at the third codon position and the created AUG is out of frame with the original CDS (**Figure 2**). Case B, a missense C>U mutation, occurs at the second codon position and the created AUG is in frame with the CDS (**Figure 2**). In cases C and D, a missense C>U mutation occurs at the first codon position and the created AUG is out of frame with the CDS (**Figure 2**). The difference between C and D is that whether the focal codon is a sense codon or a stop codon after C>U (**Figure 2**).

### 3.2 Synonymous C-to-U editing creating out-of-frame AUG is more advantageous

**Figure 3:**
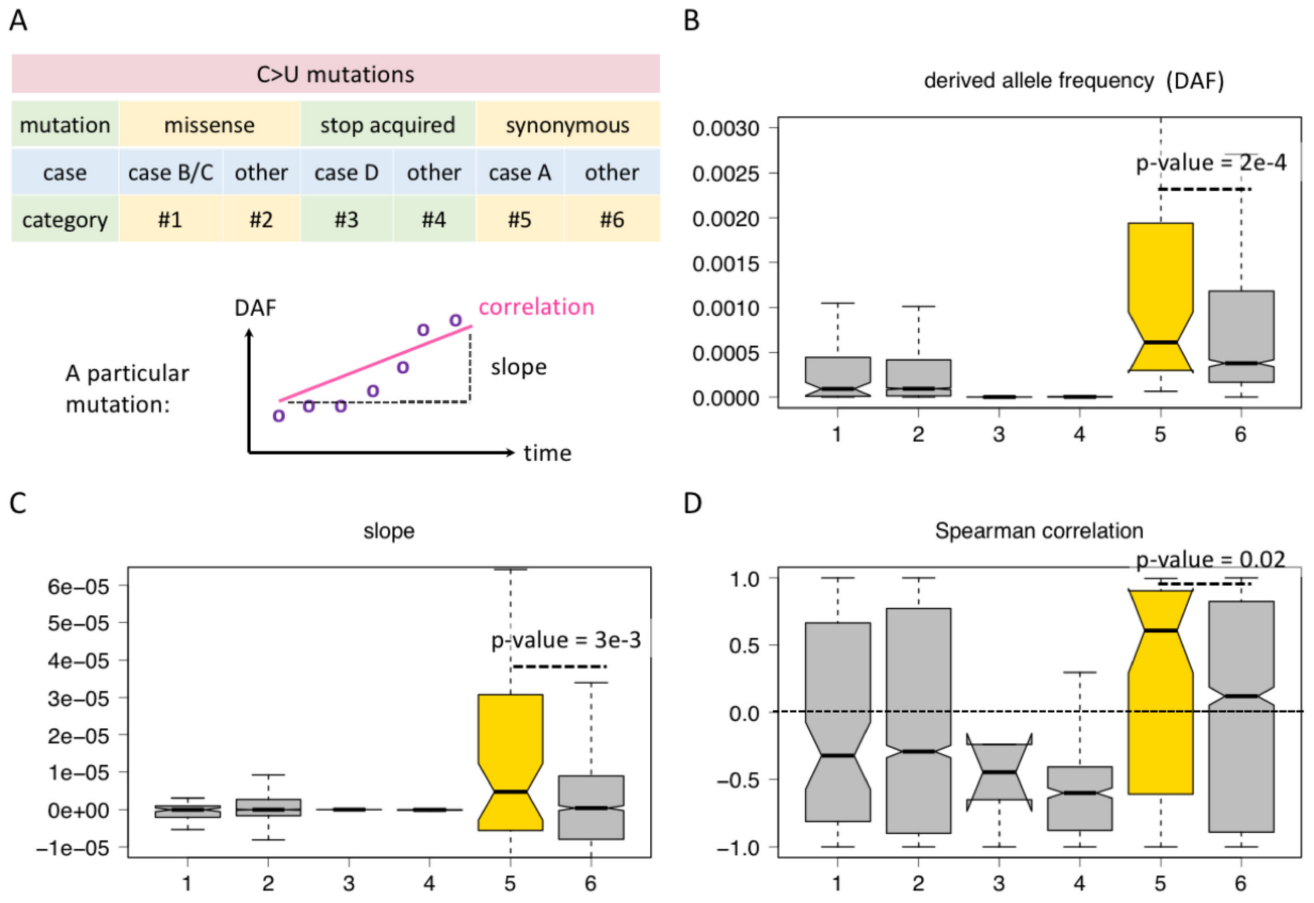
Comparison between C-to-U RNA editing sites regarding whether they create AUG. **(A)** Definition of different categories of C-to-U editing sites and parameters like derived allele frequency (DAF), slope, and correlation. **(B)** DAF of different categories of sites. KS test determined the *p*-value. **(C)** Slope of different categories of sites. KS test determined the *p*-value. **(D)** Spearman correlation coefficient of different categories of sites. KS test determined the *p*-value.

Although the four cases shown in **Figure 2** include missense, synonymous, and stop-codon acquired mutations, not all possible sequence contexts of C-to-U editing are exhausted. Here, we consider all C-to-U RNA editing sites in CDS and classified them into six categories (**Figure 3A**). For C>U missense mutations, category #1 refers to cases B/C in **Figure 2** where an AUG was created no matter whether this new AUG is in frame or out of frame. Category #2 refers to the missense mutations not belonging to category #1, which means they do not create an AUG triplet (**Figure 3A**). For stop-codon acquired mutations by C>U, such as CGA>UGA, CAG>UAG, and CAA>UAA, category #3 refers to case D in **Figure 2**, where the C>U is located in an AUG context after mutation, and category #4 is the stop-acquired mutations not belonging to category #3 (**Figure 3A**). For C>U synonymous mutations, category #5 refers to the C-to-U editing located in an AUG context after mutation, and category #6 refers to the C-to-U not within an AUG context (**Figure 3A**). In a word, the difference between categories #1 and #2, between categories #3 and #4, or between categories #5 and #6 is that the former is located in an AUG context (creates an internal AUG), but the latter is not.

Then, for each C-to-U RNA editing site, we looked at the rise and fall of its DAF and calculated the correlation and slope of DAF against different time points (Li et al., 2023). Slope is defined as *d*AF/*d*t. A correlation coefficient > 0 or a slope > 0 suggests that the DAF is increasing with time so that the mutation might be beneficial and advantageous (**Figure 3A**). If the correlation < 0 or the slope < 0, then the mutation might be deleterious. The absolute value of the correlation coefficient or slope represents the extent of the advantage/deleteriousness of the mutation.

For the six categories of C-to-U RNA editing sites, we demonstrate their DAF at the last time point of data collection (**Figure 3B**), the distribution of their slope values (**Figure 3C**), and the distribution of their Spearman correlation coefficients (**Figure 3D**). We first noticed that the overall DAF exhibits synonymous > missense > stop-acquired (**Figure 3B**), which agrees with our intuition that the stop-acquired and missense mutations are overall more deleterious than synonymous mutations. Then, we compared the different categories within each functional group.

For missense C>U sites, categories #1 and #2 do not show significant differences among these features, neither do categories #3 and #4 of stop-acquired mutations (**Figures 3B–D**). That is to say, whether the C>U mutation creates an AUG or not does not affect the global adaptiveness of missense or stop-acquired mutations. This is understandable since the effect of a missense mutation strictly depends on how the change in protein sequence will affect protein function, and so does a stop-acquired mutation. The effect of creating an internal AUG seems minor compared to the effect of changing the protein sequence.

However, for synonymous C>U sites, all the three parameters show that category #5 sites are “better” than category #6 sites (**Figures 3B–D**). The only difference between categories #5 and #6 is whether they create an internal AUG (out of frame), and this indicates that category #5 sites gain additional advantage due to the creation of AUG. This faint difference between categories #5 and #6 is only detectable in synonymous sites because synonymous mutations themselves are nearly neutral so that the selection pressure on additional factors could be observable.

### 3.3 ORFs created by synonymous C-to-U have high expressibility

So far, we only observed that the category #5 synonymous mutations creating an AUG are more advantageous than the remaining category #6 synonymous mutations (**Figures 3B–D**). However, we do not exactly know why creating an AUG is beneficial. Importantly, this difference is not caused by the change in synonymous codon preference because, here, all synonymous mutations are C-to-U, keeping the same direction of codon usage bias (if any).

**Figure 4:**
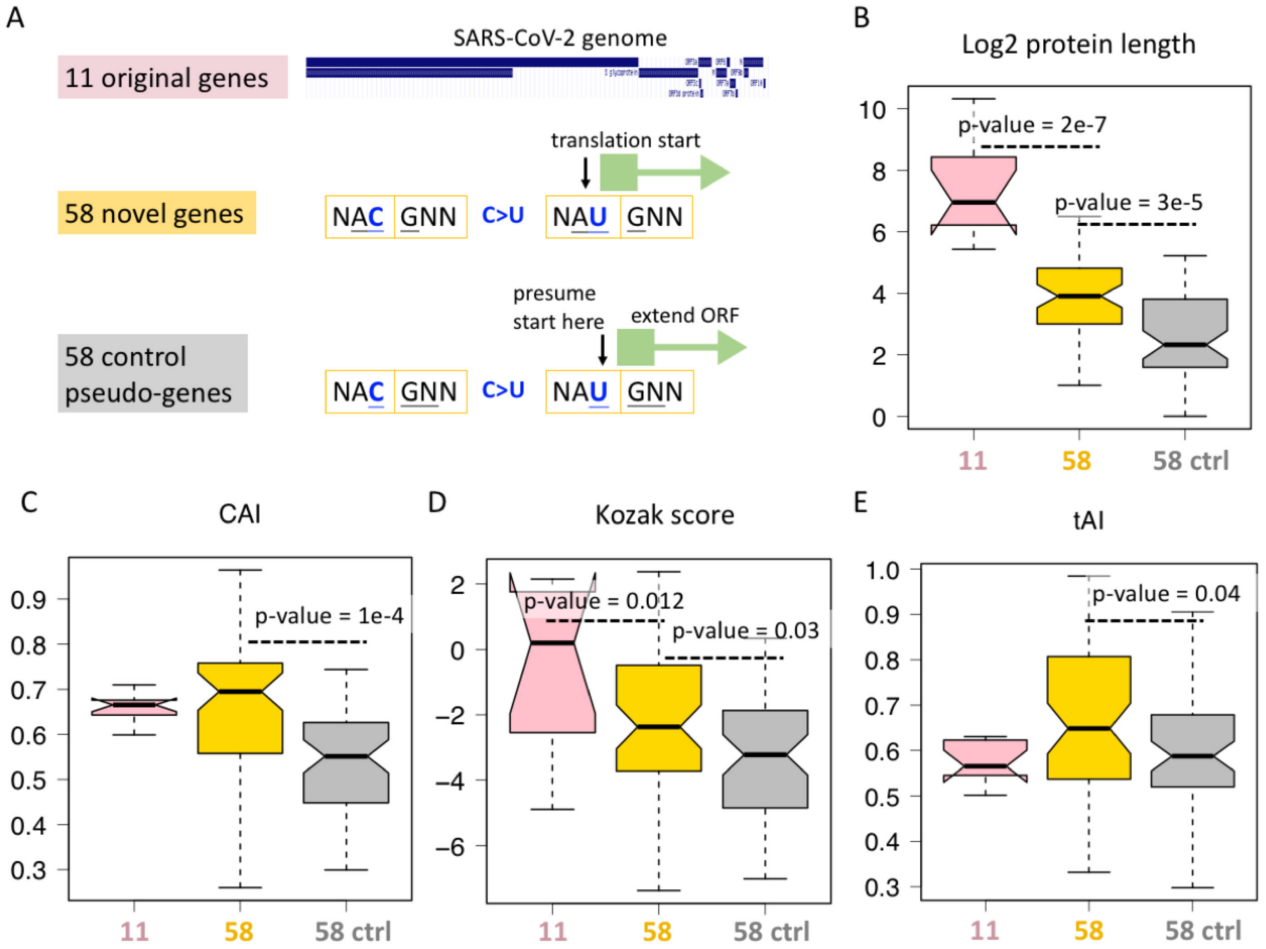
ORFs created by C-to-U RNA editing. **(A)** Definition of 11 original genes (ORFs) of SARS-CoV-2, 58 novel genes (ORFs) starting from an AUG created by synonymous C-to-U editing at the third codon position, and the corresponding 58 control genes (ORFs) starting from one nucleotide downstream the 58 synonymous C-to-U editing sites creating AUG. **(B)** Protein length of different categories of genes. KS test determined the *p*-values. **(C)** Codon adaptation index **(CAI)** of different categories of genes. KS test determined the *p*-values. **(D)** Kozak score of different categories of genes. KS test determined the *p*-values. **(E)** tRNA adaptation index (tAI) of different categories of genes. KS test determined the *p*-values.

The only possible advantage of category #5 synonymous mutations comes from the ORFs they created. We therefore compared the available features of different ORFs (**Figure 4A**). First, the SARS-CoV-2 genome has 11 non-redundant genes, corresponding to 11 ORFs, termed 11 original genes (**Figure 4A**). Then, the 58 category #5 C>U synonymous mutations create 58 AUG triplets, and each AUG will initiate a region of ORF until a stop codon is encountered. We define them as 58 novel genes (**Figure 4A**). To find a control group of ORFs, we focus on the 58 created AUGs. We presume that the reading frame does not start at the first nucleotide of AUG but at the second nucleotide of AUG (**Figure 4A**), then we extend the ORF until a stop codon is encountered. We name these 58 ORFs as “control pseudo-genes” (**Figure 4A**).

For each ORF, we measured its protein length (**Figure 4B**), CAI (**Figure 4C**), Kozak score (**Figure 4D**), and tAI (**Figure 4E**). Not surprisingly, the original 11 genes are better than the 58 novel genes in many aspects (**Figures 4B–E**). This comparison does not make new sense as it is well-known that the sequences of novel genes are less optimal than those of old genes. However, when we consider the 58 control genes, we find that the 58 novel genes are better than the corresponding control group in many ways (**Figures 4B–E**). CAI correlates with expression level, Kozak score determines translation initiation rate, and tAI affects the translation elongation rate. All these three parameters lead to a putative higher “expressibility” of 58 novel genes compared to matched controls (**Figures 4C–E**), suggesting that the C>U mutations that create these 58 novel ORFs might be the result of long-term natural selection. During evolution, mutations creating a less-optimal ORF might already be eliminated by purifying selection, and those currently observed mutations have to be beneficial in some ways. Another unexplained feature is protein/ORF length (**Figure 4B**). Although ORF length itself does not represent any adaptive features, longer ORF indeed suggests that this is more likely to be a functional gene. Usually, extending an ORF in a random sequence will encounter numerous stop codons, making the ORF very short, and only when this gene is functional can we obtain a longer ORF.

In this part (**Figure 4**), we provide genomic evidence that the 58 novel ORFs created by synonymous C-to-U RNA editing are likely to be functional, which nicely explains the observation that the 58 category #5 synonymous mutations are more advantageous than other C>U synonymous mutations (**Figure 3**).

## 4 Discussion

The C-to-U RNA editing of hosts is continuously driving the endless mutations and fast evolution of SARS-CoV-2 (Liu et al., 2022; Li et al., 2023). Deleterious mutations are suppressed and beneficial mutations are positively selected. While much attention has been paid to the missense mutations that change the viral proteins, few studies focus on the possibility that rampant C-to-U editing creates novel ORFs and how these events evolve. In our study, we focus on the synonymous C-to-U sites that change an ACG triplet to AUG, and compare these events with the remaining synonymous C-to-U sites. From the AF profile and the evolutionary tendency, we found that the former seems more beneficial than the latter, indicating an additional advantage of creating an internal AUG.

Here, only the comparison within synonymous mutations is informative because the evolution of missense mutations is largely determined by the effect of this variant on the protein function, and this strong selection pressure will mask the effect of creating an AUG. In contrast, the effect of synonymous mutation itself is much weaker than missense mutations and thus the benefit of creating an AUG could be detected. A potential effect of synonymous mutation is the change in codon optimality (Li et al., 2020b). The human genome slightly prefers G/C-ending codons so that synonymous mutations might change the codon preference. However, when we compare different categories of C-to-U RNA editing sites, their effect on codon preference should be the same.

To explain why the synonymous C-to-U sites creating AUG (58 such sites) are more advantageous than other synonymous C-to-U sites, we tried to investigate the novel ORFs created by the AUG. We found that the 58 novel ORFs have significantly higher CAI, tAI, and Kozak score than the random controls, suggesting that these 58 novel genes have higher “expressibility”, including higher expression and higher translation rate. However, considering that the predicted ORFs are not necessarily translated as many novel ORFs might be pseudogenes, we looked at the ORF length and found that 58 novel ORFs are significantly longer than random expectation. Normally, non-translated ORFs are usually short due to the encounter of stop codons, but a functional gene might be longer as they represent a small fraction of many novel genes that survive the purifying selection.

Following our finding, here comes an intuitive question whether similar mechanisms have been observed in other viral infections. We should clarify that our study is the first to report the role of C-to-U RNA editing in creating novel viral ORFs. However, similar roles of other RNA modifications have indeed been reported in non-viral species. For example, A-to-I RNA editing is prevalent in animals (Yablonovitch et al., 2017; Eisenberg and Levanon, 2018; Duan et al., 2023c; Ma et al., 2023; Zhan et al., 2023; Zhang et al., 2023; Duan et al., 2024a) and fungi (Feng et al., 2022; Duan et al., 2023b; Feng et al., 2024). The equivalence between I and G (Walkley and Li, 2017; Ma et al., 2024) makes A-to-I RNA editing able to change protein sequence and also create/alter ORFs. In insects, multiple A-to-I RNA editing events take place in the 5′UTR and can create novel small ORFs in the non-coding region according to the annotation (Yu et al., 2016; Zhang et al., 2017; Xu et al., 2023; Duan et al., 2024b; Zhao et al., 2024; Zheng et al., 2024). In cephalopods (mollusk), abundant A-to-I RNA editing directly affects the assembly and annotation of ORFs (Alon et al., 2015; Liscovitch-Brauer et al., 2017; Shoshan et al., 2021). In fungi, A-to-I RNA editing at canonical stop codons abolishes the stop codon to let the ORF extend (Qi et al., 2024). All these cases exerted by A-to-I RNA editing are related to ORF, but none of them were reported in virus.

Then, another question worth mentioning is, does this C-to-U editing regulate the integration of virus into the host genomes? So far, no direct experimental evidence supports this notion. Nevertheless, there are several indirect messages that help us judge this possibility: (1) C-to-U RNA editing by the host cells is prevalent in viral sequences, not only restricted to RNA viruses like SARS-CoV-2 (Harris and Dudley, 2015; Bian et al., 2023; Pu et al., 2023). (2) C-to-U RNA editing alters the GC content of viral sequence, leading to altered translation rates (Li et al., 2020b; Zhu et al., 2022). Since the fast translation of viral genes will facilitate the expression and replication of virus and boost the chance of successful invasion, it remains possible that C-to-U RNA editing can regulate the integration of virus to the host genomes.

## 5 Conclusion

In summary, our study proposes a possible mechanism, which is the rampant C-to-U RNA editing that leads to the emergence of *de novo* genes in SARS-CoV-2. We also provide evidence for the positive selection and high expressibility of the novel genes. Our ideas should be helpful in understanding the prevalent mutations and the evolution and adaptation of SARS-CoV-2.

## Data Availability

The original contributions presented in the study are included in the article/supplementary material. Further inquiries can be directed to the corresponding authors.
